# Identification of osteoarthritis-associated chondrocyte subpopulations and key gene-regulating drugs based on multi-omics analysis

**DOI:** 10.1038/s41598-025-90694-w

**Published:** 2025-04-11

**Authors:** Ting Hao, Zhiwei Pei, Sile Hu, Zhenqun Zhao, Wanxiong He, Jing Wang, Liuchang Jiang, jirigala Ariben, Lina Wu, Xiaolong Yang, Leipeng Wang, Yonggang Wu, Xiaofeng Chen, Qiang Li, Haobo Yang, Siqin Li, Xing Wang, Mingqi Sun, Baoxin Zhang

**Affiliations:** 1 The Second Affiliated Hospital of Inner Mongolia Medical University, Hohhot, 010050 Inner Mongolia China; 2 Tianjin Hospital, Tianjin University, Jiefang Nan Road 406, Hexi District, Tianjin, 300211 China; 3 Inner Mongolia Medical University, Hohhot, 010050 Inner Mongolia China; 4 Bayannur City Hospital, Bayannur City, 015000 Inner Mongolia China; 5 Aier Eye Hospital, Tianjin University, No. 102 Fukang Road, Tianjin, 300000 China; 6 Sanya People’s Hospital, No. 558 Jiefang Road, Sanya City, Hainan Province China; 7 Baotou Medical College Bayannur Clinical Medical College, Bayannur City, 015000 Inner Mongolia China

**Keywords:** Osteoarthritis, Bioinformatics, Machine learning, Single cell analysis, Molecular docking, Cell biology, Drug discovery, Molecular biology, Diseases, Medical research

## Abstract

The mechanism by which chondrocytes respond to mechanical stress in joints significantly affects the balance and function of cartilage. This study aims to characterize osteoarthritis-associated chondrocyte subpopulations and key gene targets for regulatory drugs. To begin, single-cell and transcriptome datasets were obtained from the Gene Expression Omnibus (GEO) database. Cell communication and pseudo-temporal analysis, as well as High-dimensional Weighted Gene Co-expression Network Analysis (hdWGCNA), were conducted on the single-cell data to identify key chondrocyte subtypes and module genes. Subsequently, Consensus Cluster Plus analysis was utilized to identify distinct disease subgroups within the osteoarthritis (OA) training dataset based on the key module genes. Furthermore, differential gene expression analysis and GO/KEGG pathway enrichment analysis were performed on the identified subgroups. To screen for hub genes associated with OA, a combination of 10 machine learning algorithms and 113 algorithm compositions was integrated. Additionally, the immune and pathway scores of the training dataset samples were evaluated using the ESTIMATE, MCP-counter, and ssGSEA algorithms to establish the relationship between the hub genes and immune and pathways. Following this, a network depicting the interaction between the hub genes and transcription factors was constructed based on the Network Analyst database. Moreover, the hub genes were subjected to drug prediction and molecular docking using the RNAactDrug database and AutoDockTools. Finally, real-time fluorescence quantitative PCR (RT-qPCR) was employed to detect the expression of hub genes in the plasma samples collected from osteoarthritis patients and healthy adults. In the OA sample, there is a significant increase in the proportion of prehypertrophic chondrocytes (preHTC), particularly in subgroups 6, 7, and 9. We defined these subgroups as OA_PreHTC subgroups. The OA_PreHTC subgroup exhibits a higher communication intensity with proliferative-related pathways such as ANGPTL and TGF-β. Furthermore, two OA disease subgroups were identified in the training set samples. This led to the identification of 411 differentially expressed genes (DEGs) related to osteoarthritis, 2485 DEGs among subgroups, as well as 238 intersecting genes and 5 hub genes (MMP13, FAM26F, CHI3L1, TAC1, and CKS2). RT-qPCR results indicate significant differences in the expression levels of five hub genes and their related TFs in the clinical blood samples of OA patients compared to the healthy control group (NC). Moreover, these five hub genes are positively associated with inflammatory pathways such as TNF-α, JAK-STAT3, and inflammatory response, while being negatively associated with proliferation pathways like WNT and KRAS. Additionally, the five hub genes are positively associated with neutrophils, activated CD4 T cell, gamma delta T cell, and regulatory T cell, while being negatively associated with CD56dim natural killer cell and Type 17T helper cell. Molecular docking results reveal that CAY10603, Tenulin, T0901317, and Nonactin exhibit high binding activity to CHI3L1, suggesting their potential as therapeutic drugs for OA. The OA_PreHTC subgroups plays a crucial role in the occurrence and development of osteoarthritis (OA). Five hub genes may exert their effects on OA through interactions with PreHTC cells, other chondrocytes, and immune cells, playing a role in inhibiting cell proliferation and stimulating inflammation, thus having high diagnostic value for OA. Additionally, CAY10603, Tenulin, T0901317, and Nonactin have potential therapeutic effects for OA patients.

## Introduction

Osteoarthritis (OA) is common in middle-aged and older people, characterized by degenerative changes in the morphology, composition, and mechanical properties of joint cartilage, leading to chronic pain, joint instability, stiffness, and joint deformity^1^. OA can affect any joint in the body, with the knee joint being the most commonly affected, making knee osteoarthritis the fourth leading cause of disability worldwide^2,3^. Currently, the main treatment for early OA is focused on relieving pain and reducing joint wear and tear, while advanced OA patients often require total knee replacement surgery^4^. Therefore, clarifying the pathogenesis of osteoarthritis, identifying new target molecules involved in the pathophysiological processes of osteoarthritis, is crucial for the prevention and treatment of this disease.

The knee joint as a weight-bearing joint experiences continuous mechanical loading, and the cartilage is the only tissue that cushions this load on the joint surface. Furthermore, the chondrocytes are the exclusive cell type in the articular cartilage tissue^5^. The mechanisms by which chondrocytes respond to mechanical loading in the joint have a significant impact on the balance and function of the cartilage^6^. Mechanical loading not only causes “wear and tear” but also serves as a driving force to activate cellular signaling and promote the production of pro-inflammatory mediators and degradative enzymes, thus promoting chondrocyte phenotype shifts and the development of osteoarthritis^7^. Moreover, single-cell RNA sequencing (scRNA-seq) technology has demonstrated the presence of distinct chondrocyte subpopulations that variably drive inflammation in osteoarthritis (OA) patients^8^.

The single-cell RNA sequencing (scRNA-seq) technique has been widely used to detect a variety of omics data at the single-cell level. It provides a more in-depth description of cell diversity and states, helps to understand cell heterogeneity, and can discover new cell types and clinical biomarkers^9^. The analysis of scRNA-seq offers the opportunity for comprehensive and precise characterization of cell and molecular features in both healthy and diseased tissues. Identifying potential disease-dependent differences at the single-cell level can greatly aid in understanding molecular mechanisms and identifying target cells and pathways^10^. Combining the discovery of disease targets through scRNA-seq with molecular docking studies based on network pharmacology may provide guidance for the development of effective strategies for the prevention and treatment of osteoarthritis (OA).

In this study, we integrated bioinformatics, machine learning, and network pharmacology to analyze single-cell and transcriptome data, aiming to explore the subtypes of chondrocytes related to osteoarthritis and identify key gene regulatory drugs. We utilized multiple algorithms to construct a network of hub genes’ relationships with immunity and pathways, as well as their interactions with transcription factors and drugs. Our work has the potential to optimize precision therapy and improve early detection and treatment of OA.

## Materials and methods

### Data acquisition

We obtained single-cell and transcriptome datasets from the Gene Expression Omnibus (GEO) database (https://www.ncbi.nlm.nih.gov/geo/). Specifically, the dataset GSE152805^11^, which comprises single-cell sequencing data of human osteoarthritis (OA) synovial tissue and chondrocytes from the medial load-bearing, pathologic region of OA knee joints, was utilized. Additionally, transcriptional datasets including GSE19060^12^, GSE82107^13^, and GSE89408^14^, which cover various OA and normal control samples, were also downloaded. The GSE152805 dataset encompasses 3 OA cartilage tissue samples and 3 healthy cartilage tissue samples, generated on the GPL20301 platform. GSE19060 includes 5 OA samples and 3 normal controls (NC) samples from the GPL570 platform. GSE82107 comprises 10 OA samples and 7 NC samples, also on the GPL570 platform, whereas GSE89408 contains 22 OA samples and 28 NC samples from the GPL11154 platform.These datasets include both RNA-seq data (used as the training set) and microarray data (used as validation sets). All datasets used in this study involve human samples. For data integration, the GSE89408 and GSE82107 datasets were merged without batch effects using the sva^15^ package in R. This merged dataset served as the primary training set, whereas GSE19060, GSE82107, and GSE89408 were used as validation sets. For each transcriptome dataset, gene symbols were converted from probes using built-in annotation files, and the data were normalized by applying a log + 1 transformation.

### Data quality control

To ensure the reliability and accuracy of the analyses, rigorous data quality control (QC) measures were implemented for each dataset:

Single-Cell Dataset (GSE152805).

1. Filtering Low-Quality Cells: Cells with < 1000 unique molecular identifiers (UMIs) or > 10% mitochondrial gene expression were excluded.
2. Batch Effect Correction: Batch effects were mitigated using the sva^15^ package in R to ensure consistency across different sequencing runs.
3. Normalization: Data were normalized using the Seurat normalization method to adjust for library size and sequencing depth differences.

Transcriptome Datasets (GSE19060, GSE82107, GSE89408).

1. Normalization: Data were normalized using the Robust Multi-array Average (RMA) method to account for technical variability.
2. Outlier Detection: Outliers were identified using principal component analysis (PCA) and samples with extreme values were removed.
3. Batch Effect Correction: Batch effects were addressed using the ComBat method^16^ to align the distributions of the datasets.

### Processing of single-cell data

The percentages of mitochondria, ribosomes, and red blood cells were calculated using the Percentage Feature Set function. Genes with expression levels exceeding 250 were selected, while mitochondrial gene expression accounted for less than 5%. The DecontX^16^ tool was then applied to eliminate ambient RNA contamination. Subsequently, the merged ScRNA-seq data underwent normalization, and the top 2000 highly variable genes were identified using the Find Variable Feature function. All genes were standardized using the Scale Data function, and the selected 2000 highly variable genes were subjected to dimensionality reduction using the Run PCA function. Batch correction was performed using the Find Integration Anchors function, followed by cell clustering using the “Find Neighbors” and “Find Cluster” functions to identify cell clusters. Next, further dimensionality reduction was carried out using Run UMAP. Subsequently, cell subgroups were identified using the Find All Markers function, with a Minpct value of 0.25 (minimum differential gene expression ratio). Cell subtype annotation was based on previous literature^17^, and the slingshot R package^18^ was utilized for pseudo-temporal analysis of specific cell subtypes.

It is worth noting that the definition of chondrocyte subpopulations was based on cellular gene expression patterns, clustering analysis results, and comparison with known marker genes for chondrocyte subtypes. By integrating these pieces of information, we identified distinct chondrocyte subtypes, including (list subtype names), ensuring that the defined subpopulations are both statistically significant and biologically meaningful.

### Differential gene expression analysis

To identify key genes associated with OA, we performed differential gene expression (DGE) analysis separately on the RNA-seq training set and the microarray validation sets. For the RNA-seq data, DGE analysis was conducted using DESeq2 in R. For the microarray data, we employed the limma package in R. This allowed us to identify genes that were differentially expressed between OA and normal control samples within each dataset type. The results from these analyses were then integrated in subsequent steps to ensure the robustness of our findings.

### CellChat analysis

CellCall^19^ is a dataset that collects ligand-receptor-transcription factor (L-R-TF) axis data based on the KEGG pathway. It serves as a tool for inferring intercellular communication networks and internal regulatory signals by integrating intracellular and intercellular signals. We utilized cellcall to further elucidate the specific pathways between high and low scoring groups and other cell subtypes.

For single-cell data and cell classification, we performed cell communication analysis using R package CellChat (V1.6.0)^20^. CellChatDB.human, an internal database within CellChat, was utilized as a reference for analyzing interactions between cells and investigating the relationships of 32 pathways among cells.

### SCENIC analysis

The R package SCENIC^21^ was used to analyze the regulatory networks of transcription factors (TFs) and genes within cell subgroups. Enrichment analysis was conducted using the gene-motif rankings from the RcisTarget database with the hg19-tss-centered-10 kb data, to detect the regulatory networks of transcription start sites (TSS) and genes within different subgroups. Visualization was performed using the pheatmap package.

### High dimensional WGCNA

The high-dimensional Weighted Gene Co-expression Network Analysis (hdWGCNA) is commonly used for analyzing co-expression networks in single-cell RNA sequencing (scRNA-seq)^22^. hdWGCNA is highly modular and can construct co-expression networks across multiple scales of cellular and spatial hierarchies. It can identify candidate biomarker genes or therapeutic targets based on the connectivity within gene sets and their association with phenotypes. We used hdWGCNA to analyze the selected modules and identify key module genes for downstream analysis.

### Functional enrichment analysis

Gene Ontology (GO) analysis is a common method for conducting large-scale functional enrichment studies, including biological process (BP), molecular function (MF), and cellular component (CC). The Kyoto Encyclopedia of Genes and Genomes (KEGG)^23^ is a widely used database that stores information regarding genomes, biological pathways, diseases, and drugs. Utilizing the R package clusterProfiler^24^, differential gene expression is subjected to GO annotation analysis and KEGG pathway enrichment analysis, with an FDR threshold of *p* < 0.05 considered to have statistical significance.

To investigate the critical biological processes associated with hub genes, we conducted gene set enrichment analysis (GSEA) as a method to assess whether a specific gene set exhibits statistical differences between two biological states^25^. We obtained the gene set “c2.cp.kegg.v7.1.entrez.gmt” from the MSigDB database and analyzed the biological functions of each hub genes. A significance level of *p*.adjust < 0.05 was considered indicative of enrichment. Furthermore, in order to examine the differences in biological processes between the high and low vascular mimicry scores groups, we employed the GSVA package for enrichment analysis^26^. We downloaded the gene set “c2.cp.kegg.v7.5.1.symbols.gmt” from the MSigDB database for GSVA analysis, with a significance level of *p*.adjust < 0.05 considered to indicate enrichment.

### Consensus cluster plus analysis

Consistency clustering^27^ is a method that provides quantitative evidence for determining the number and members of possible clusters in a data set. We conducted Consensus Cluster Plus analysis using the genes obtained from hdWGCNA. Initially, we determined the optimal number of clusters using the R package factoextra^28^. Then, we used the k-means clustering method to perform unsupervised clustering on all patients in the training set based on the optimal number of clusters, resulting in a final classification of samples into 2 groups. Additionally, we utilized the same R package to evaluate the final clustering results. The gene expression patterns in the two sample groups were visualized using volcano plots.

### Central analysis approach

To identify the key chondrocyte subtypes and module genes associated with osteoarthritis (OA), we employed a central analysis approach combining cell communication analysis, pseudo-temporal analysis, and High-dimensional Weighted Gene Co-expression Network Analysis (hdWGCNA).

Cell Communication and Pseudo-Temporal Analysis: Using the R package CellChat, we analyzed the communication networks between different chondrocyte subtypes to understand their interactions. Pseudo-temporal analysis was conducted using the slingshot R package to infer the differentiation trajectories among chondrocyte subtypes.

High-dimensional Weighted Gene Co-expression Network Analysis (hdWGCNA): hdWGCNA was utilized to construct co-expression networks from the single-cell RNA-seq data. This analysis helped in identifying gene modules that are highly co-expressed and functionally related. The optimal soft threshold was determined based on the scale-free topology model fit index.

### Machine learning algorithms and their combinations

To develop robust diagnostic models, we integrated multiple machine learning algorithms and their permutations.

Machine Learning Algorithms: We employed a total of 10 machine learning algorithms, including Random Survival Forest (RSF), Elastic Net (Enet), Lasso, Ridge, Stepwise Cox, CoxBoost, Cox Partial Least Squares Regression (plsRcox), Supervised Principal Components (SuperPC), Generalized Boosted Regression Model (GBM), and Survival Support Vector Machine (survival-SVM).

Algorithm Combinations: To enhance model performance, we tested 113 different combinations of the above algorithms. The comprehensive algorithm combinations were applied to fit predictive models based on the leave-one-out cross-validation (LOOCV) framework.

Model Selection and Validation: The performance of each model was evaluated using the Harrell’s concordance index (C-index) in the training and validation datasets. The model with the highest average C-index across all validation datasets was selected as the optimal model for further analysis.

### The relationship between the hub genes and the immune and signaling pathways

We used the ESTIMATE algorithm to assess stromal and immune cell scores in the organization based on expression data^30^. The ESTIMATE algorithm was obtained from the public website (https://sourceforge.net/projects/estimateproject/) and is capable of estimating stromal and immune scores based on specific biomarkers associated with stromal and immune cell infiltration in the samples, thereby predicting the levels of stromal and immune cells in the tissue, and subsequently calculating the correlation of key genes with them. Additionally, we employed the MCP-counter method^31^, which allows for robust quantification of the absolute abundance of eight immune cell types (T cells, CD8 T cells, Cytotoxic lymphocytes, B lineage, NK cells, Monocytic lineage, Myeloid dendritic cells, Neutrophils) and two stromal cell types in heterogeneous tissue from transcriptomic data. Subsequently, we calculated the correlation of key genes with these cell types.

The Gene Set Variation Analysis (GSVA) is a non-parametric, unsupervised method for gene set enrichment, which estimates scores for pathways or markers based on transcriptome data^26^. We used the ssGSEA method from the R package GSVA^26^ , referenced from PMID: 28052254^32^, to obtain the genes for 28 immune cells. In addition, we obtained 50 HALLMARK pathways from the h.all.v7.5.symbols.gmt file on the GSEA website, and calculated pathway scores for the samples using the ssGSEA method, then we assessed the correlation of key genes with these pathways.

### Multidimensional network analysis of hub genes

Transcription factors (TFs) can regulate gene expression by interacting with target genes at the transcription stage. We constructed a regulatory network of hub genes and TFs using the Network Analyst database (https://www.networkanalyst.ca/)^33^. Additionally, we built a regulatory network of hub genes and drugs based on the RNAactDrug database (http://bio-bigdata.hrbmu.edu.cn/RNAactDrug/). We conducted docking experiments and calculated binding free energies using AutoDockTools (version 1.5.6), and visualized the final results using PyMOL (version 2.2) software^34^.

### Real-time fluorescence quantitative PCR (RT-qPCR)

Peripheral blood of five clinical osteoarthritis patients and five healthy adults were obtained from the Second Affiliated Hospital of Inner Mongolia Medical University. (This study was performed in line with the principles of the Declaration of Helsinki. Approval was granted by the Ethics Committee of Second Affiliated Hospital of Inner Mongolia Medical University. The ethical review number: YKD202002055). 5 ml peripheral venous blood was collected with EDTA-K2 anticoagulant blood collection tube. After centrifugation at 1500 r/min for 15 min, the uppermost plasma was obtained. Total RNA was extracted from plasma samples. The genomic DNA was removed from the RNA sample, and RNA was reverse transcribed using the PrimeScript™ RT reagent Kit with gDNA WIPER (R123-01, Vazyme, China). Real-Time quantitative PCR was performed using the SYBR® Premix Ex Taq (Q111-02, Vazyme, China) kit using a real-time PCR machine (ABI-7500, Applied Biosystems, USA). The PCR amplification was carried out for a total of 42 cycles. The mean + standard error of three independent experiments were calculated, with each experiment repeated three times. Relative mRNA expression levels were calculated using GAPDH as an internal reference. Primers for every gene can be found in Table 1.

**Table 1:** Information on RT-qPCR primers.

Primer name	Primer sequences (5′–3′)
GAPDH F	TGACTTCAACAGCGACACCCA
GAPDH R	CACCCTGTTGCTGTAGCCAAA
CHI3L1 F	CAGTGGGTAGGATACGACG
CHI3L1 R	TTGATGGCATTGGTGAGA
CKS2 F	CGGCATGTTATGTTACCC
CKS2 R	CTCCTCCACTCCTCTTCA
FAM26F F	ATCTGTCACCCGATGCCTAT
FAM26F R	GCGAGCCCTCAAAGAAAC
MMP13 F	CGACTTCTACCCATTTGA
MMP13 R	ACTACTTGTCCAGGTTTCA
TAC1 F	CTGAATTACTGGTCCGACTG
TAC1 R	AGAACTGCTGAGGCTTGG
CREB1 F	ATCTGTCACCCGATGCCTAT
CREB1 R	GCGAGCCCTCAAAGAAAC
CREB3 F	GTCAGAAGTGCCGAAAGA
CREB3 R	GAAGAGGTCAGGGAATGG
HDAC6 F	ACCGCTACGAGCAGGGTA
HDAC6 R	CTGGTTCCAAGGCACATT
NFKB1 F	AGCACGACAACATCTCATT
NFKB1 R	GGACCTTGCCCTTCTTGTT
RELA F	GGGGACTACGACCTGAATG
RELA R	GGGCACGATTGTCAAAGAT

### Statistical analysis

All data processing and analysis were conducted using R software (version 4.1.1). Independent Student t-tests were used to estimate the statistical significance for the comparison of two groups of continuous variables, with normal distribution. The differences between non-normally distributed variables were analyzed using the Mann–Whitney U test. The statistical significance between two groups of categorical variables was assessed using either the chi-square test or Fisher’s exact test. Pearson correlation analysis was employed to calculate the correlation coefficients between different genes. The comparison of mean values between two groups of samples utilized t-tests, while ANOVA tests were used for comparing mean values among multiple groups. All p-values were two-tailed, and statistical significance was defined as *p* < 0.05.

## Results

### Single-cell landscape analysis of osteoarthritis

**Fig. 1:**
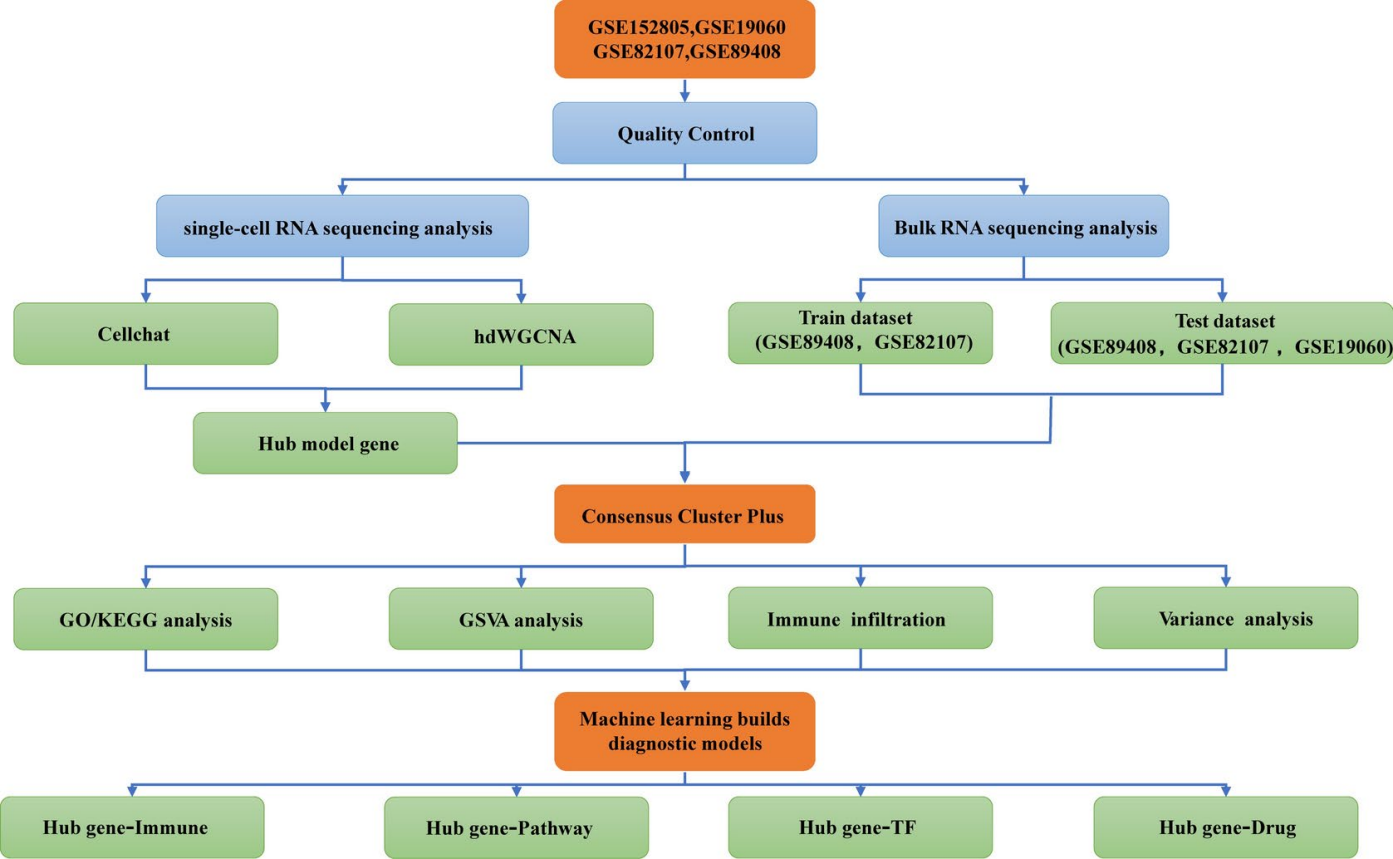
Technology roadmap.

**Fig. 2:**
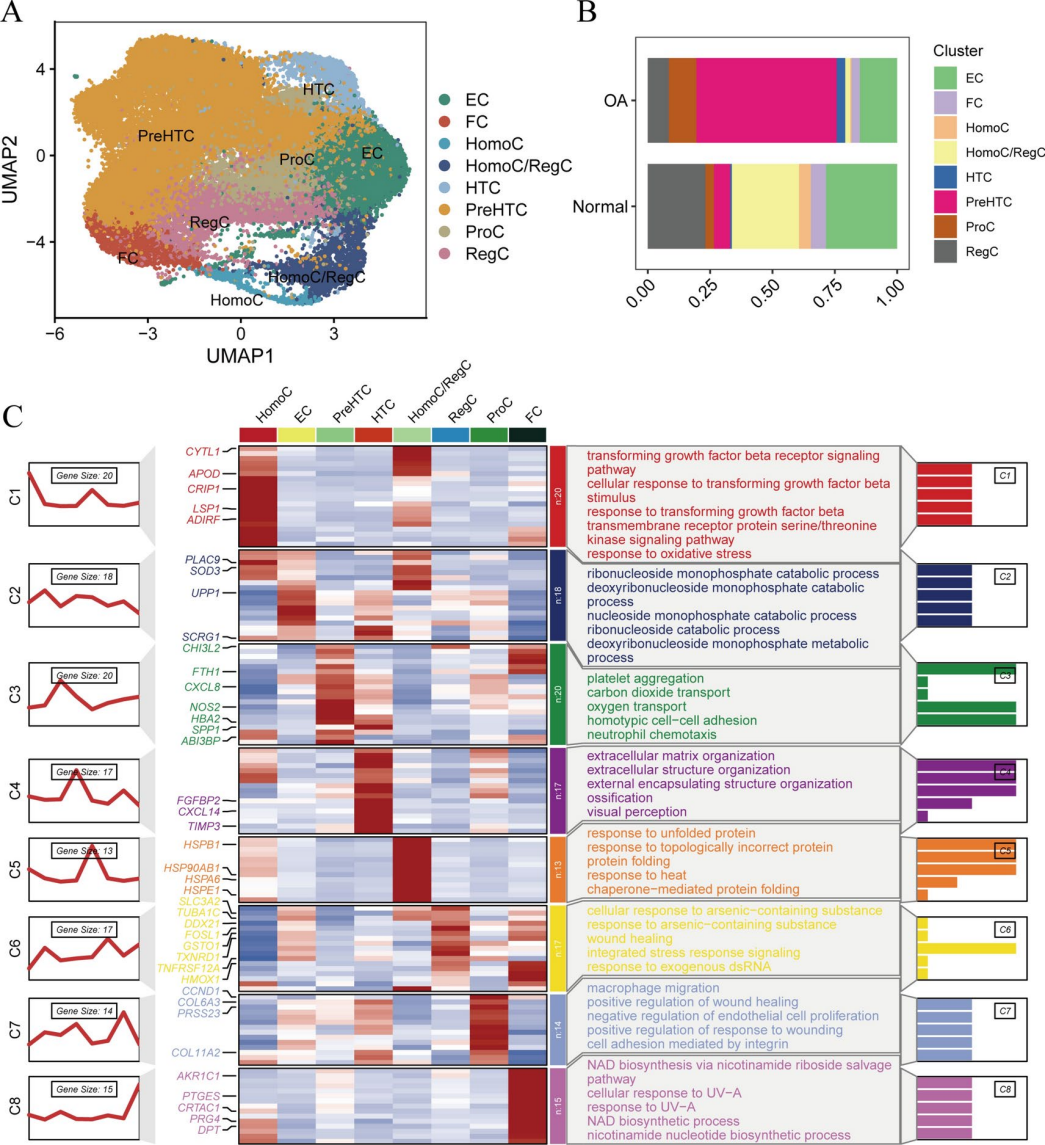
Single-cell landscape analysis of osteoarthritis. (**A**) UMAP plots after cellular annotation of the osteoarthritis single-cell dataset (GSE152805); (**B**) Cellular proportions of the eight chondrocyte subpopulations in osteoarthritic and normal articular cartilage tissues; (**C**) Analysis of key marker genes and functional enrichment of the eight chondrocyte subpopulations.

To clearly demonstrate the specific workflow of this study, Fig. 1 outlines the bioinformatics analysis process. Annotation of the single-cell data GSE152805 resulted in the identification of 8 distinct subtypes of chondrocytes: Effector chondrocyte (EC), Fibrocartilage chondrocyte (FC), Prehypertrophic chondrocyte (preHTC), Proliferative chondrocyte (ProC), Hypertrophic chondrocyte (HTC), Homeostatic chondrocyte (HomC), Regulatory chondrocyte (RegC), and Homeostatic chondrocyte/Regulatory chondrocyte (HomC/ RegC) (Fig. 2A). Visualization of the proportions of chondrocyte subtypes in normal cartilage (NC) and osteoarthritis (OA) tissues revealed a significant increase in the proportion of PreHTC and a notable decrease in the proportion of RegC in the OA tissue (Fig. 2B). Additionally, the pseudo-time analysis indicated that bone joint cartilage EC occupied the starting point, while PreHTC resided in an intermediate branch, suggesting that PreHTC may serve as a transitional cell state in chondrocyte differentiation (Supplementary Fig. 1A). Functional enrichment analysis revealed that PreHTC is mainly enriched in platelet aggregation, oxygen transport, and neutrophil chemotaxis. In contrast, the low expression of RegC is mainly enriched in repair-related functions such as wound healing and integrated stress response signaling (Fig. 2C). These data indicate functional impairments in OA cartilage tissue.

### Classification and pseudotime analysis of pre-HTC

**Fig. 3:**
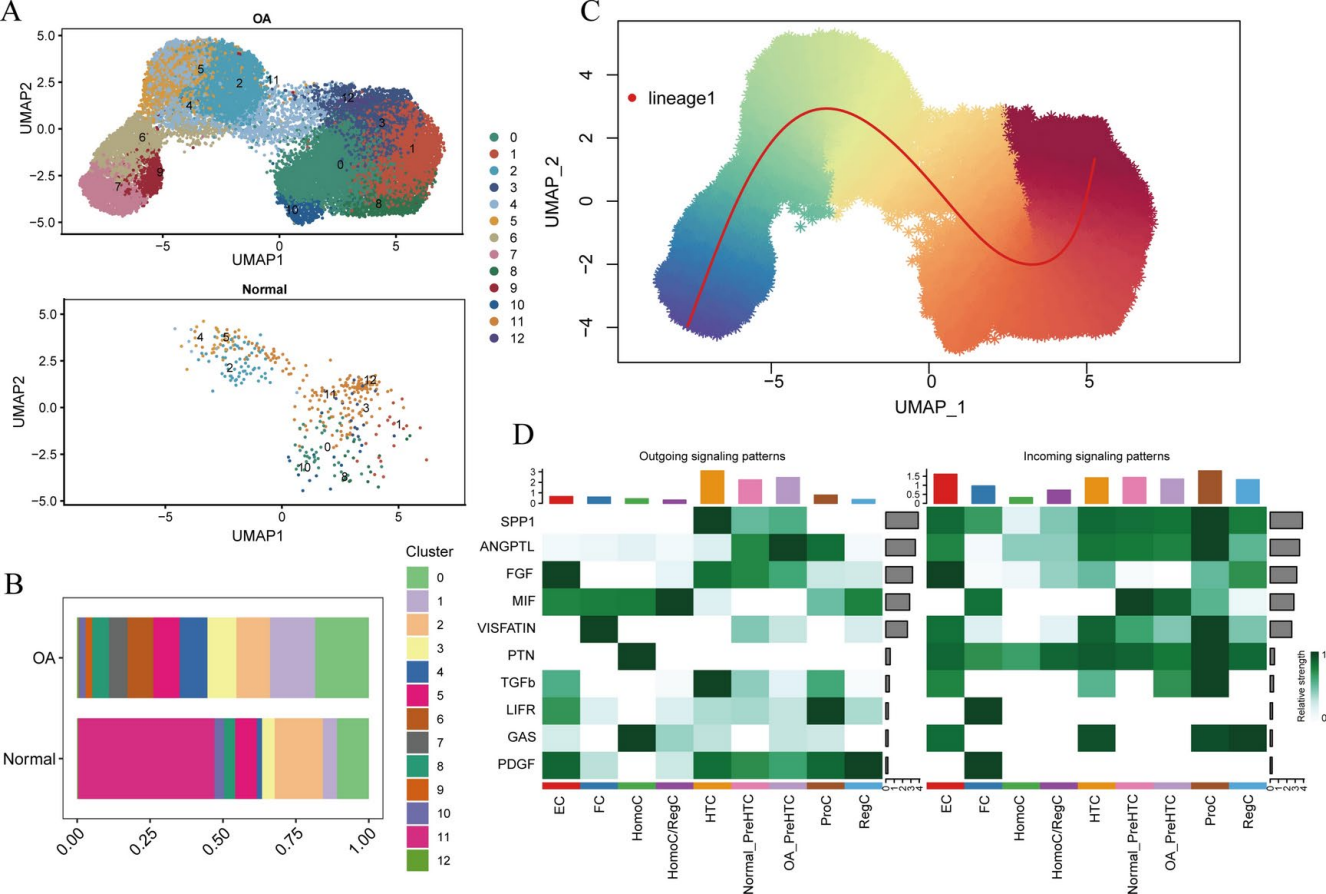
Classification of pre-HTC cells and pseudo-temporal analysis. (**A**) UMAP plot of cluster distribution difference between PreHTC in OA and Normal cartilage tissues; (**B**) Histogram of cluster distribution difference between PreHTC in OA and Normal cartilage tissues; (**C**) Pseudo-temporal differentiation trajectory map from Normal_PreHTC to OA_PreHTC; (**D**) Cell communication heatmap from Normal_PreHTC to OA_PreHTC.

After extracting PreHTC, we performed dimension reduction clustering, resulting in 13 subgroups. Our analysis based on tissue origin revealed that, in comparison to the PreHTC cell subgroups from normal cartilage tissue, 6, 7, and 9 subgroups were additionally present in OA cartilage tissue (Fig. 3A,B). So we defined the subpopulations of PreHTC cells 6, 7, and 9 in OA cartilage tissue as OA_PreHTC subpopulations, while the other subpopulations of PreHTC cells in OA cartilage tissue were defined as Normal_PreHTC subpopulations. Subsequently, we conducted pseudotime analysis to demonstrate the differentiation trajectory from Normal_PreHTC to OA_PreHTC (Fig. 3C). Furthermore, Cytotrace analysis indicated that OA_PreHTC exhibited higher levels of stemness (Supplementary Fig. 1C), suggesting that OA PreHTC may harbor potential drug targets and possess therapeutic potential. To further elucidate the functional differences between Normal_PreHTC and OA_PreHTC, we performed analysis of cellular communication. The results showed that OA_PreHTC displayed higher communication strength in pathways such as ANGPTL and TGF-β, both of which are associated with proliferative function (Fig. 3D).

**Fig. 4:**
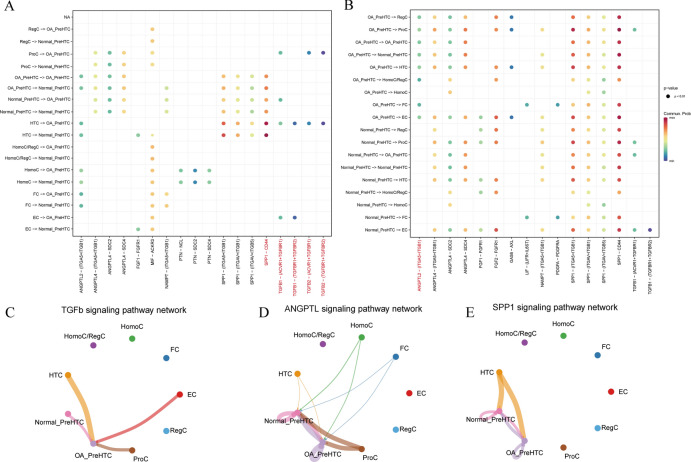
Cell communication analysis. (**A**) Cell communication results diagrams for OA_PreHTC and Normal_PreHTC as receptors; (**B**) Cell communication results diagrams for OA_PreHTC and Normal_PreHTC as ligands; (**C**) Cell communication network diagram for OA_PreHTC and Normal_PreHTC in the TGF-β signal; (**D**) Cell communication network diagram for OA_PreHTC and Normal_PreHTC in the ANGPTL signal; (**E**) Cell communication network diagram for OA_PreHTC and Normal_PreHTC in the SPP1 signal.

In addition, we conducted a cell communication analysis based on input and output signals. The results showed that when OA_PreHTC acts as a signal receptor, there is a significant TGF-β signaling pathway interaction with ProC and HTC (Fig. 4A). When OA_PreHTC functions as a ligand, significant expression of the ANGPTL (ITGA5 + ITGB1) signal receptor is observed in other cell subgroups (Fig. 4B). The cell communication network diagram indicates that OA_PreHTC primarily acts as a signal receptor in the TGF-β signal in other cells, suggesting the critical role of TGF-β signaling in OA_PreHTC (Fig. 4C). Additionally, the expression of the ANGPTL and SPP1 signals is observed in both OA_PreHTC and Normal_PreHTC (Fig. 4D–E).

### Functional enrichment analysis of OA_PreHTC and Normal_PreHTC

**Fig. 5:**
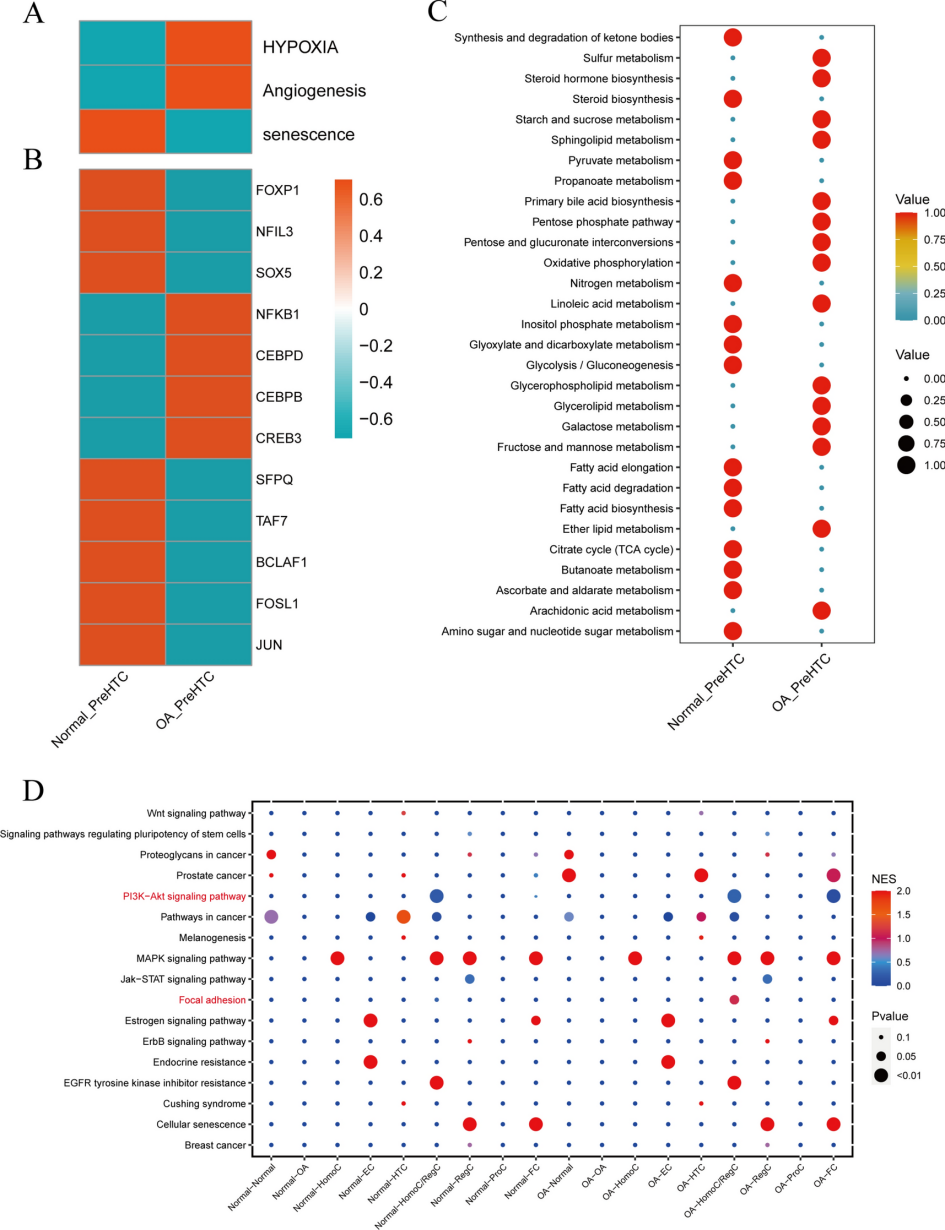
Functional enrichment analysis of OA_PreHTC and Normal_PreHTC. A. Enrichment analysis of HYPOXIA, Angiogenesis, and Senescence signaling pathways in OA_PreHTC and Normal_PreHTC. B. SCENIC analysis in OA_PreHTC and Normal_PreHTC. C. Differential cell metabolism in OA_PreHTC and Normal_PreHTC. D. Enrichment analysis of Cellcall functions.

First, we conducted functional enrichment analysis to examine the differential expression of the HYPOXIA, Angiogenesis, and Senescence signaling pathways in OA_PreHTC and Normal_PreHTC. The results revealed a significant increase in the expression levels of HYPOXIA and Angiogenesis in OA_PreHTC, whereas Senescence exhibited a significantly higher expression level in Normal_PreHTC (Fig. 5A). This indicates the presence of oxidative stress, hypoxia, and neovascularization in the articular cartilage of OA, with OA_PreHTC showing a significant proliferative state, while Normal_PreHTC displayed higher expression levels of Senescence. Subsequent SCENIC analysis identified elevated expression of transcription factors such as NFKB1, CEBPD, CEBPB, and CREB3 in OA_PreHTC, with the CEBPB signaling pathway primarily associated with neutrophil generation and cell proliferation (Fig. 5B). Additionally, metabolic differential analysis indicated a significant increase in glycolysis and oxidative phosphorylation pathways in OA_PreHTC, possibly closely related to the inflammatory environment in osteoarthritic cartilage (Fig. 5C). Finally, pathway analysis using Cellcall demonstrated a high expression of pathways such as cell adhesion between OA_PreHTC and FC, representing an important factor in the pathological processes of osteoarthritis (Fig. 5D).

### The analysis of hdWGCNA in OA_PreHTC

**Fig. 6:**
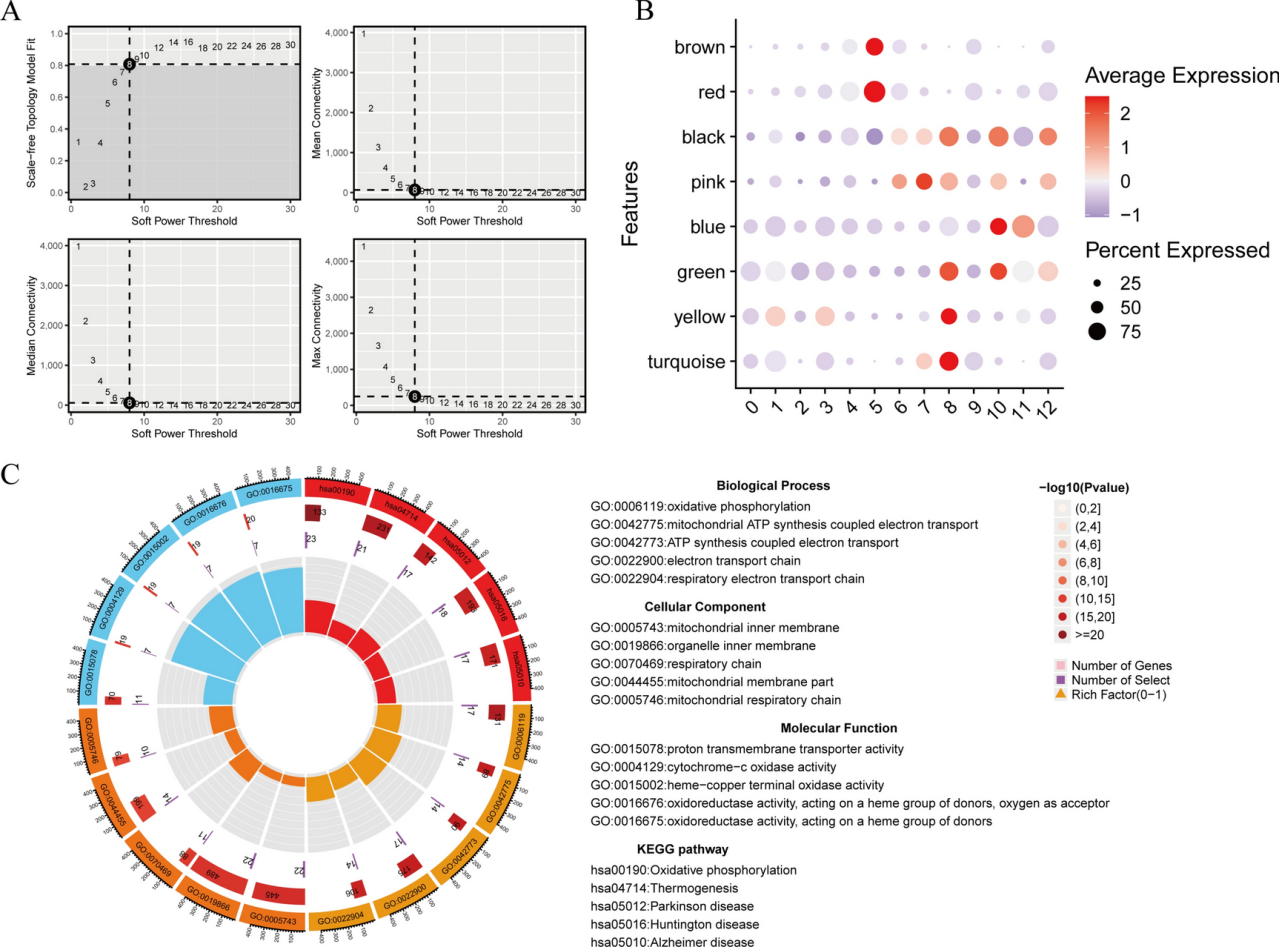
The analysis of hdWGCNA in OA_PreHTC. (**A**) Soft threshold screen for hdWGCNA analysis; (**B**) Black and pink modules significantly enriched in 6, 7, and 9clusters; C. GO/KEGG enrichment analysis of black and pink module genes.

In the study described above, we followed the extraction of PreHTC and subsequently conducted dimensionality reduction clustering, which resulted in the emergence of 13 subgroups. Initially, we carried out hdWGCNA analysis, setting the soft threshold to 8 based on the outcomes (Fig. 6A). Further scrutiny of the findings revealed a significant enrichment of the black and pink modules within clusters 6, 7, and 9 (OA_PreHTC) (Fig. 6B) (Supplementary Fig. 1D). Additionally, a noteworthy positive correlation was observed between the black and pink modules (Supplementary Fig. 1E). Subsequently, the genes associated with the black and pink modules were extracted, identifying a total of 147 genes. Functional enrichment analysis results indicated a predominant enrichment in oxidative phosphorylation and oxidoreductase activity, suggesting that oxidative stress acts as the primary factor mediating the pathogenesis of OA_PreHTC (Fig. 6C).

### Consensus cluster plus analysis

**Fig. 7:**
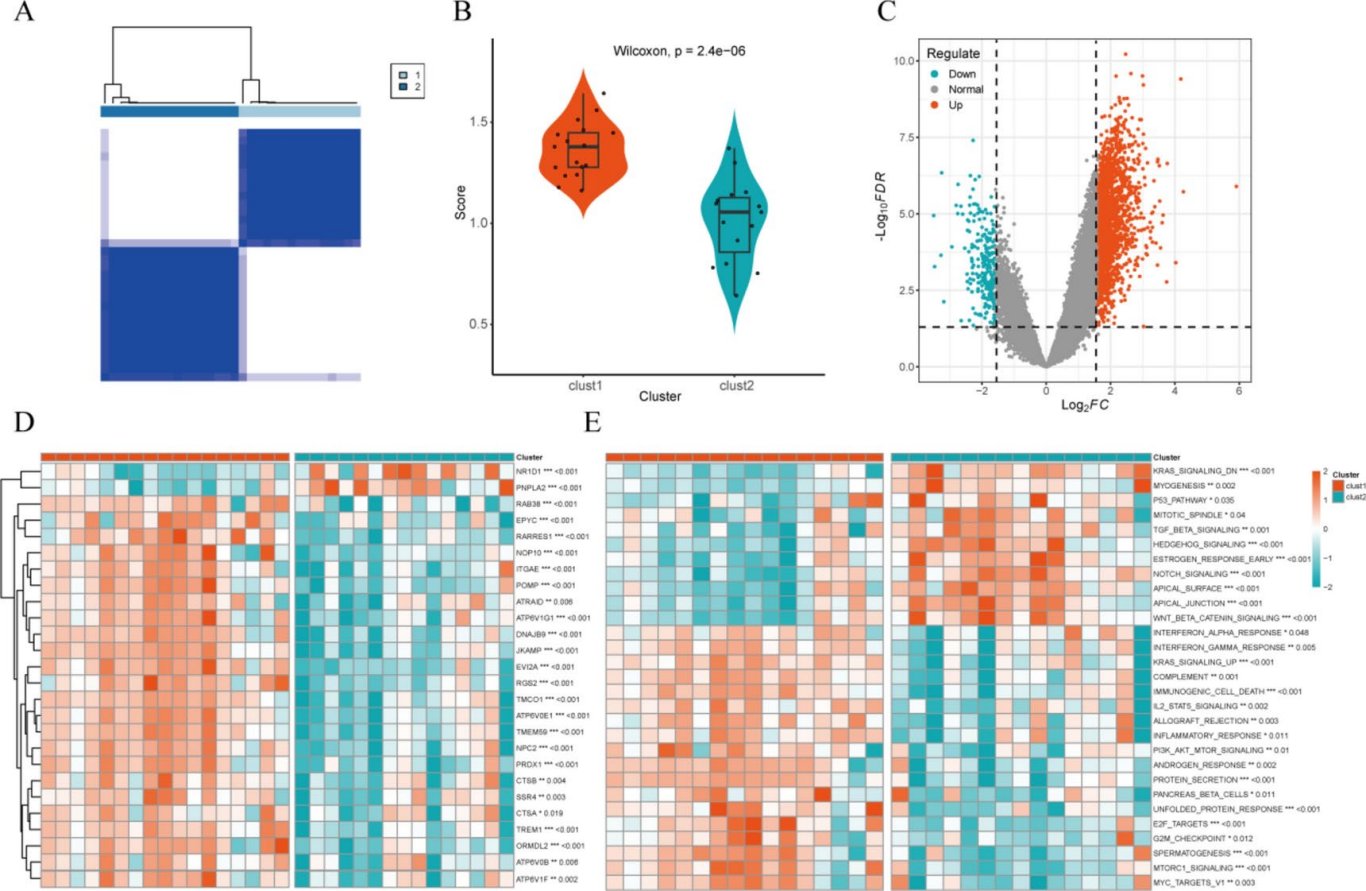
Consensus Cluster Plus analysis. (**A**) Classification of OA diseased samples from the training set into cluster1 (n = 17) and cluster2 (n = 15) by consistent cluster analysis; (**B**) Differences in expression levels of black and pink module genes in clusters; (**C**) Volcano map of differentially expressed genes between clusters; (**D**) Distribution of differentially expressed genes between clusters; (**E**) GSVA pathway enrichment analysis between clusters.

To identify molecular subtypes based on black and pink module genes, we conducted Consensus Cluster Plus analysis using OA diseased samples from the training set. After considering the cumulative distribution function (CDF) curve and Delta area, we selected k = 2 as the unique and non-overlapping number of subtypes (Fig. 7A).

Subsequently, the genes associated with the black and pink modules were extracted, resulting in a total of 147 genes. Notably, our analysis revealed that 238 of these module genes overlapped with the differentially expressed genes (DEGs) identified in our previous study. This overlap indicates a strong consistency in the identification of OA-related genes across different methodologies. Functional enrichment analysis further suggested that oxidative stress, particularly through oxidative phosphorylation and oxidoreductase activity, plays a crucial role in the pathogenesis of OA_PreHTC.

To further elucidate the molecular mechanisms underlying OA, we performed a comprehensive analysis of DEGs. As shown in Supplementary Fig. 2, we identified 411 key DEGs that were consistently differentially expressed across multiple datasets and subgroups. These 411 genes were then subjected to logistic and Lasso analysis to select a final set of diagnostic marker genes (Supplementary Fig. 2E-F; note: this analysis is distinct from the previous gene selection process that identified the 9 biomarker genes).

We assessed the expression of module genes within the two subtypes, revealing significantly higher expression of module genes in cluster1, suggesting a potential higher correlation of cluster1 with OA_PreHTC (Fig. 7B,D). Additionally, we identified 2458 differentially expressed genes between the two subtypes, with │log2FC│ > 1.5 and adj.*P*.value < 0.05 (Fig. 7C). Furthermore, to elucidate the biological functions of the differentially expressed genes, we conducted Gene Set Variation Analysis (GSVA). The results indicated a close association of cluster1 with inflammatory pathways, KRAS pathway, STAT pathway, and PI3K-AKT pathway, while cluster2 was primarily associated with NOTCH and WNT pathways (Fig. 7E).

Moreover, to further refine our understanding of the key genes involved, we intersected the GSE89408’s OA and Normal differentially expressed genes with those between the subgroups, yielding 238 key DEGs (Supplementary Fig. 2C,D). This step was followed by gene selection based on logistic and Lasso analysis, which ultimately led to the identification of 9 biomarker genes: BAIAP3, CHI3L1, CKS2, FAM26F, IBSP, MIR31HG, MMP13, SNORA74A, and TAC1 (Supplementary Fig. 2E,F; note: these 9 genes are a subset of the previously mentioned 411 key DEGs).

### Machine learning builds diagnostic models

**Fig. 8:**
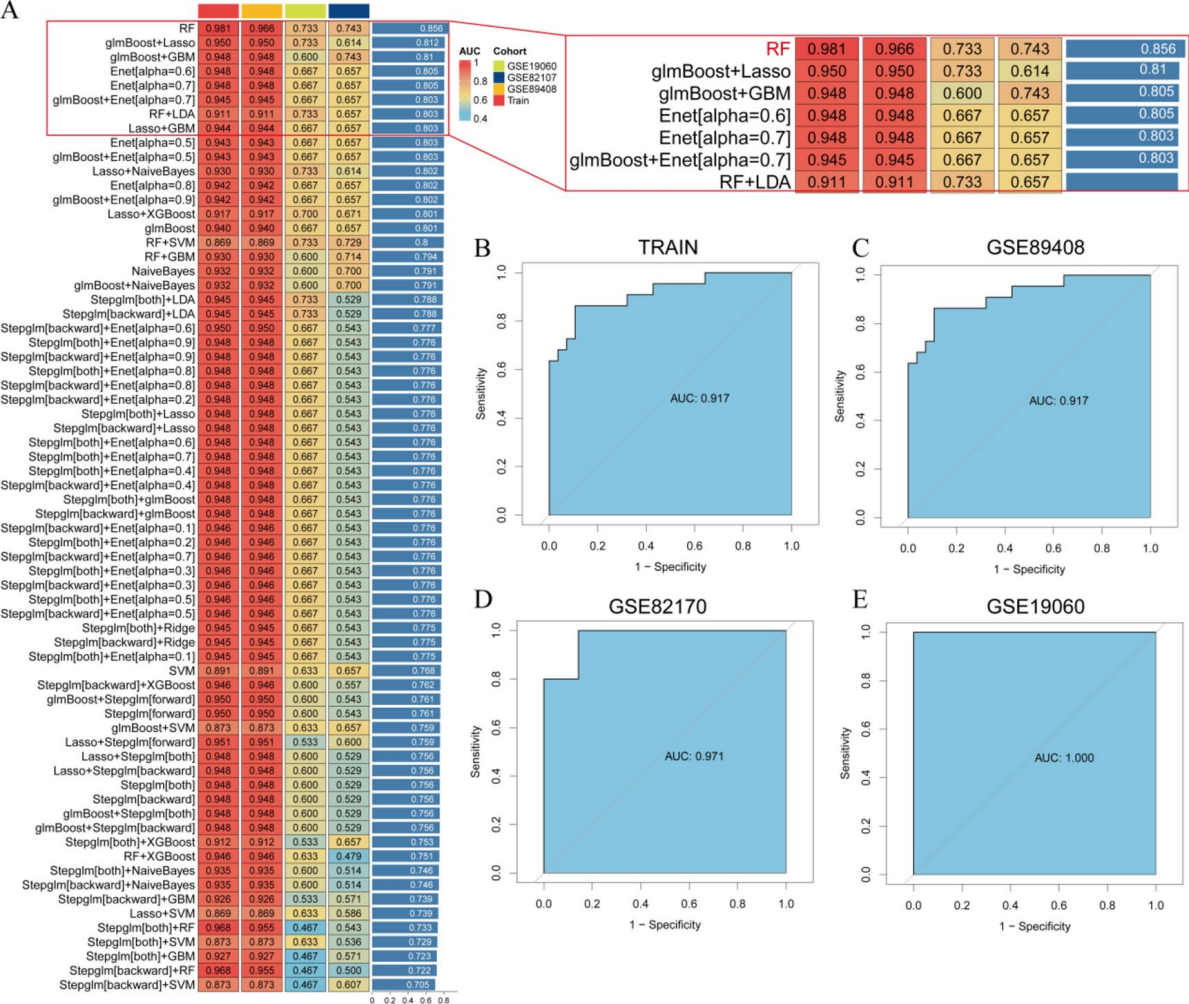
Screening hub genes through machine learning. (**A**) Validation efficacy of models evaluated based on different combinations of machine learning algorithms; (**B**) AUC curves for the training set; (**C**) AUC curves for the validation set GSE89408; (**D**) AUC curves for the validation set GSE82107; (**E**) AUC curves for the validation set GSE19060.

We integrated the 9 diagnostic marker genes and evaluated 10 machine learning algorithms along with 113 algorithm combinations to select the optimal diagnostic model based on AUC and c-index comparisons. After considering both the number of genes and model validation performance, we determined that the key gene set derived from Random Forest (RF) calculation achieved the highest c-index and AUC values (Fig. 8A). Subsequently, we validated the model’s accuracy using the training set and additional validation sets, and the results consistently indicated a high level of accuracy (Fig. 8B–E).

Furthermore, based on the RF calculation, we pinpointed 5 hub genes: MMP13, FAM26F, CHI3L1, TAC1, and CKS2. Although FAM26F did not exhibit differential expression in the pseudotime analysis specifically focused on the OA_PreHTC subgroups, its inclusion as a hub gene in our study was justified by several compelling reasons. Firstly, FAM26F emerged as one of the top candidate genes through our comprehensive machine learning analysis, which integrated multiple algorithms and datasets, ensuring a robust selection process not solely reliant on a single type of analysis. Secondly, FAM26F has been implicated in immune regulation and inflammation, processes that are intimately linked to OA pathogenesis ^48^. Given its potential biological significance in OA and its strong presence in our machine learning models, we included FAM26F in the subsequent drug-database screen to explore its therapeutic potential.

These hub genes were found to be significantly associated with the OA_PreHTC subgroups that we had identified in our scRNA-seq data, particularly subgroups 6, 7, and 9. While they primarily correlated with the OA_PreHTC subpopulations, further analysis is necessary to fully elucidate their potential associations with other preHTC subpopulations, such as Normal_PreHTC, and to understand their broader roles in OA pathogenesis.

### The relationship between the hub genes and the immune and pathway

**Fig. 9:**
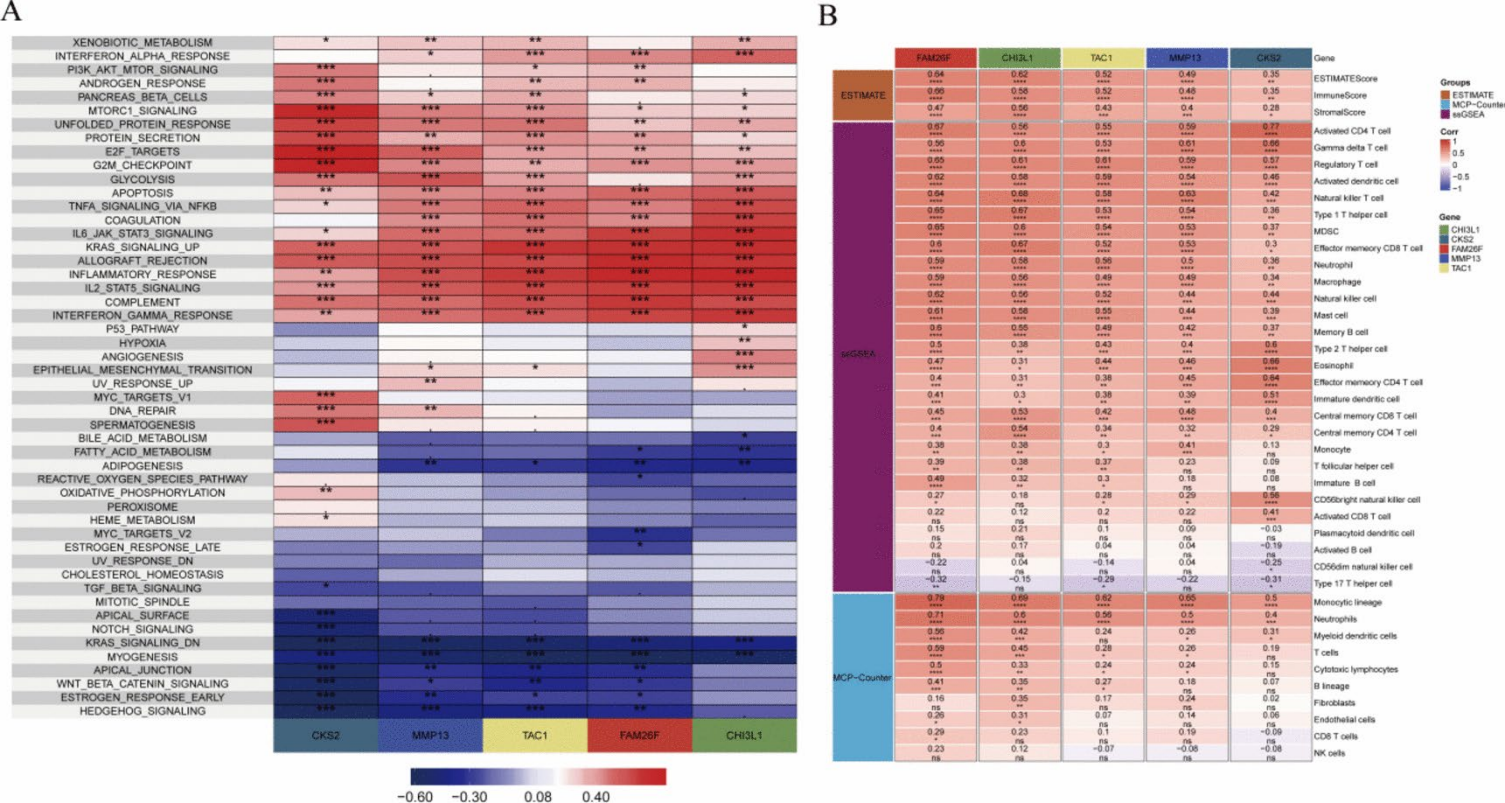
The relationship between the hub genes and the immune and pathway. (**A**) Correlation of hub genes with pathways in the training set; (**B**) Correlation of hub genes with immune scores in the training set. (*in the figure represents *P* < 0.05, **represents *P* < 0.01, ***represents *P* < 0.001, ****represents *P* < 0.0001, and ns represents *P* > 0.05).

We obtained 50 pathway files (h.all.v7.5.symbols.gmt) of the HALLMARK from the GSEA^25^ website and evaluated the scores of each pathway in the training set using the ssGSEA method. Subsequently, we separately calculated the Pearson correlation between our hub genes and the scores of these pathways and visually represented the results. The findings indicated that all five hub genes exhibited a positive correlation with inflammatory pathways such as TNF-α, JAK-STAT3, and inflammatory responses, while displaying a negative correlation with proliferation pathways like WNT and KRAS (Fig. 9A). These data suggest that the five hub genes may exert their effects in osteoarthritis by inhibiting cell proliferation and stimulating inflammation. Additionally, we evaluated the immune scores of the training set using the ESTIMATE and MCP-counter methods. Subsequently, we obtained the genes of 28 immune cells from a reference (PMID:28052254)^32^ and evaluated the scores of each sample using the ssGSEA method. Following this, we calculated the Pearson correlation between our hub genes and these immune scores separately and represented them visually. The results indicated that all five hub genes exhibited a positive correlation with immune cells such as Neutrophils, Activated CD4 T cell, Gamma delta T cell, and Regulatory T cell, while showing a negative correlation with CD56dim natural killer cell and Type 17T helper cell (Fig. 9B).

### Analysis of the hub genes interaction network

**Fig. 10:**
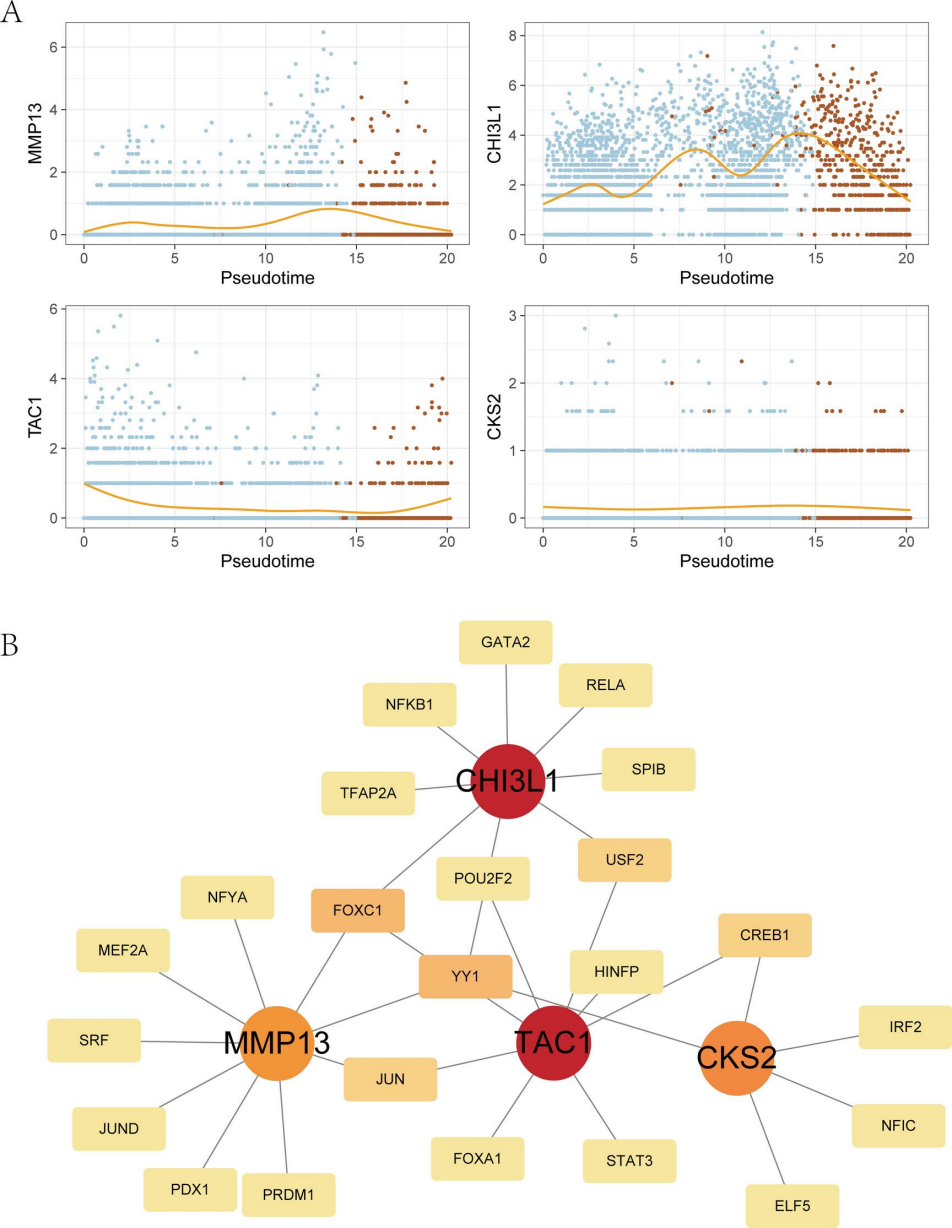
Interaction network analysis of TF-hub genes. (**A**) The pseudo-temporal analysis of hub genes; (**B**) The regulatory network of hub genes and TFs.

Subsequently, we conducted a pseudo-temporal analysis to visualize the expression levels of 5 hub genes, and identified expression changes in 4 genes during the differentiation process from Normal_PreHTC to OA_PreHTC. Particularly, the expression of CHI3L1 exhibited the most significant variation, with an increase during the differentiation from Normal_PreHTC to OA_PreHTC, followed by a rapid decrease upon completion of differentiation, suggesting its potential mediation role in the differentiation process (Fig. 10A). To further elucidate the regulatory roles of hub genes in the pathogenesis of OA, we constructed TF-hub genes regulatory networks for the 4 hub genes using the NetworkAnalyst database (https://www.networkanalyst.ca/)^33^. The results revealed a close association between CHI3L1 and NFKB1, while CSK2 was found to be closely linked with CREB1 (Fig. 10B).

**Fig. 11:**
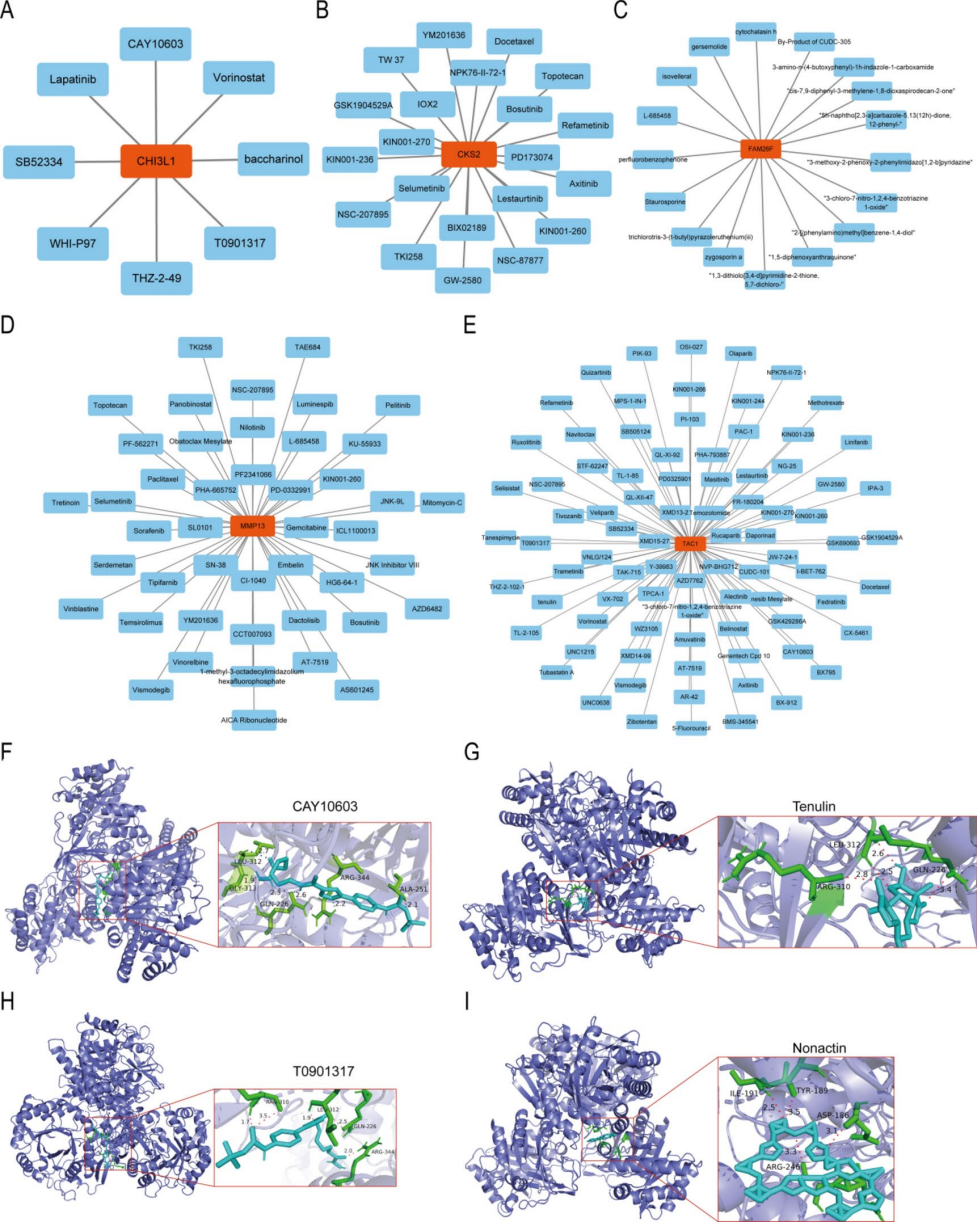
Interaction network analysis of hub genes-Drug. (**A**–**E**) Drug-hub gene regulatory networks were constructed for each of the five hub genes based on the RNAactDrug database. The selection parameters were FDR < 0.01, *P*-value < 0.001, and the data sources were CCLE, GDSC and Cellminer; (**F**–**I**) Molecular docking pattern map of CHI3L1 with drugs. The free energy of binding of CHI3L1 to CAY10603 was − 4.75 kcal/mol (**F**), the free energy of binding of CHI3L1 to Tenulin was − 6.55 kcal/mol (**G**), the free energy of binding of CHI3L1 to T0901317 was − 4.86 kcal/mol (**H**), the free energy of binding of CHI3L1 to The free energy of CHI3L1 binding to T0901317 was − 4.86 kcal/mol (**H**), and that of CHI3L1 binding to Nonactin was − 14.1 kcal/mol (**I**). In the figure, purple color represents CHI3L1, cyan color is the drug small molecule, green color is the amino acid residue, and red color is the hydrogen bond that binds them.

In addition, based on the RNAactDrug database, Drug-hub gene regulatory networks were constructed for 5 hub genes (Fig. 11A–E). Subsequently, we used AutoDockTools-1.5.6 software to dock the core components of the drugs with the CHI3L1 target protein for molecular validation. Additionally, we utilized PyMOL 2.2 software to visualize the docking patterns of some well-binding small molecule ligands with the target protein (Fig. 11F–I). The results indicate that CAY10603, Tenulin, T0901317, and Nonactin exhibit high binding activity to CHI3L1, suggesting their potential as therapeutic drugs for OA.

### The expression level of the hub genes in patients with clinical osteoarthritis

**Fig. 12:**
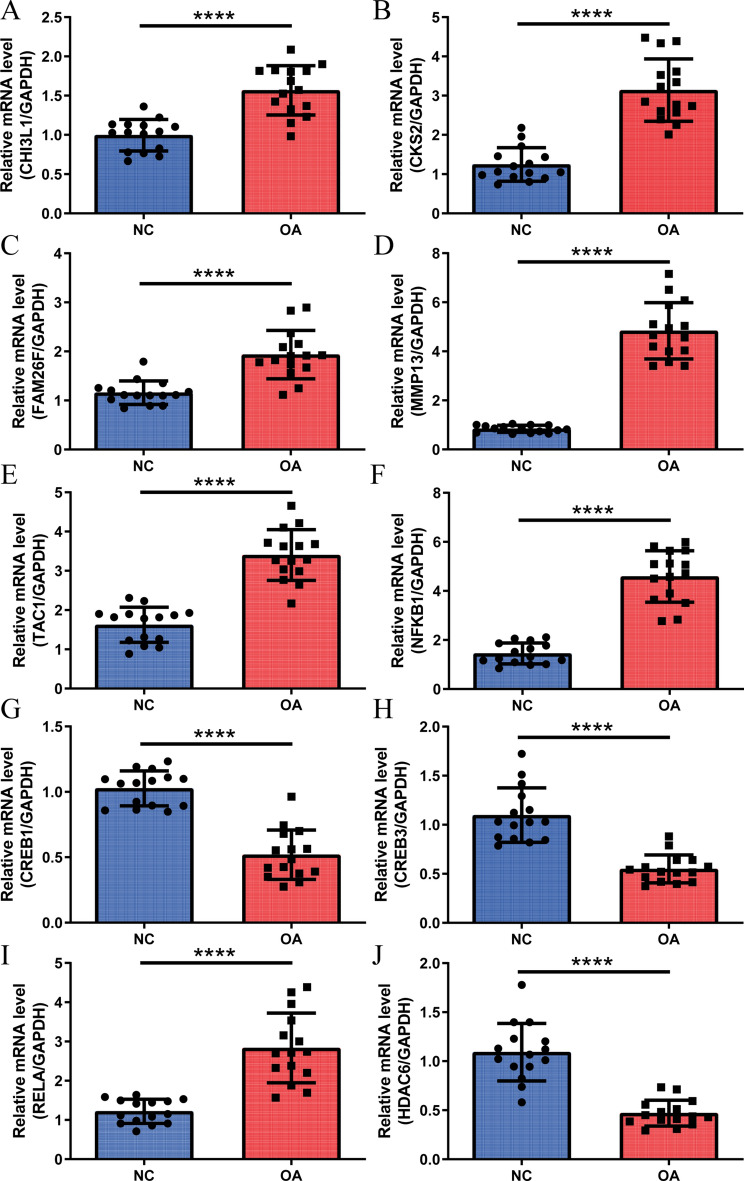
The molecular expression levels of Hub genes and their associated TF were measured in clinical blood samples using RT-qPCR. (**A**–**E**) The mRNA expression levels of five hub genes (CHI3L1, CKS2, FAM26F, MMP13, and TAC1) in plasma of NC and OA groups; (**F**–**I**). The mRNA expression levels of hub genes-associated TFs (NFKB1, CREB1, CREB3, and RELA) in plasma of NC and OA groups; (**J**) The mRNA expression levels of HDAC6, a CAY10603 target, in plasma of NC and mRNA expression levels of HDAC6, a target of CAY10603, in plasma of OA group. (****P < 0.0001, vs. Control, N = 5, n = 3).

To explore the expression patterns of Hub genes and their associated transcription factors (TFs), clinical peripheral blood samples were collected from both the normal control group (NC) and the osteoarthritis group (OA). The mRNA expression levels of five hub genes (CHI3L1, CKS2, FAM26F, MMP13, and TAC1) in plasma were measured (Fig. 12A–E), along with the mRNA expression levels of hub genes-related TFs (NFKB1, CREB1, CREB3, and RELA) (Fig. 12F–I), and the mRNA expression level of the HDAC6 target of CAY10603 (Fig. 12J). The RT-qPCR results revealed a significant upregulation in the mRNA expression levels of CHI3L1, CKS2, FAM26F, MMP13, TAC1, NFKB1, and RELA in the OA group compared to the NC group, while the mRNA expression levels of CREB1, CREB3, and HDAC6 were significantly decreased (Fig. 12A–J).

## Discussion

An increasing number of studies indicate that OA is a systemic musculoskeletal disease involving activation of the innate and adaptive immune systems and accompanied by inflammation^35^. However, the pathophysiological mechanisms of osteoarthritis are still not fully understood, and finding effective therapeutic drugs remains a challenge. In-depth research on the pathogenesis and treatment targets of OA is crucial for its management.

The study utilized cell communication, pseudo-temporal analysis, and hdWGCNA analysis to examine single-cell data and identify subgroups 6, 7, and 9 of preHTC as key chondrocyte subgroups in osteoarthritis (OA). These subgroups were collectively defined as OA_PreHTC subgroups. Notably, the OA_PreHTC subgroups exhibited heightened communication intensity with proliferative pathways such as ANGPTL and TGF-β. Following this, dimension reduction clustering and hdWGCNA analysis were performed to identify critical modules within the OA_PreHTC subgroups. Subsequently, transcriptional data were analyzed using an integrated approach involving 10 machine learning algorithms, 113 algorithm combinations, and Consensus Cluster Plus analysis, leading to the identification of two distinct diseased subgroups, 238 intersecting differentially expressed genes, and 5 hub genes. Furthermore, various algorithms including ESTIMATE, MCP-counter, and ssGSEA were employed to establish the relationships between differentially expressed genes among subgroups, critical module genes, and hub genes, with immune function and pathways. The findings underscored the significant role of the OA_PreHTC subgroups in the onset and progression of osteoarthritis. The 5 hub genes were implicated in modulating cellular proliferation, inflammation, and interactions between PreHTC cells, other chondrocytes, and immune cells, imparting considerable diagnostic value for OA. Furthermore, compounds such as CAY10603, Tenulin, T0901317, and Nonactin may hold potential in targeting CHI3L1 for the treatment of OA patients.

Although these datasets represent cartilage tissues from OA patients, there may exist certain differences in gene expression profiles across them due to factors such as sample collection methods, tissue processing protocols, and patient variability. Recognizing these potential variations is crucial for interpreting our findings and ensuring the robustness of our conclusions.

To mitigate the impact of dataset-specific biases, we employed rigorous bioinformatics approaches, including batch effect correction and normalization steps. Furthermore, we validated our findings using multiple validation datasets, which strengthened the reliability of our results. However, it is important to note that subtle differences in gene expression patterns could still persist after these adjustments.

For instance, variations in the severity of OA, the specific joint involved (e.g., knee vs. hip), and the duration of disease could lead to distinct transcriptional signatures across datasets. Additionally, demographic factors such as age, gender, and ethnicity of the patient populations may also contribute to the observed gene expression differences.

Despite these potential variations, our study consistently identified prehypertrophic chondrocytes (PreHTC) as a key chondrocyte subpopulation involved in OA pathogenesis across all analyzed datasets. The identification of five hub genes (MMP13, FAM26F, CHI3L1, TAC1, and CKS2) and their association with inflammatory pathways and immune cells further validates the robustness of our findings. Future studies could explore these genes in more homogeneous patient populations to better understand their specific roles in OA development and progression.

First, our analysis indicates that the 5 hub genes are positively correlated with inflammatory pathways such as TNF-α, JAK-STAT3, and inflammatory response, while they are negatively correlated with proliferation pathways such as WNT and KRAS. Previous studies have shown that Chitinase 3-like 1 (CHI3L1) and Matrix Metallopeptidase 13 (MMP13) serve as pro-inflammatory biomarkers, associated with tissue remodeling and bone resorption^36,37^. For example, Song et al.^37^ demonstrated that in a mouse model of osteoarthritis, the expression levels of CHI3L1 and MMP13 are positively correlated with inflammatory factors such as TNF-α, IL-1β, and IL-6, and can be suppressed by overexpression of nuclear factor erythroid 2-related factor 2 (Nrf2). Tachykinin Precursor 1 (TAC1) encodes four products of the tachykinin peptide family, namely substance P, neurokinin A, neurokinin K, and neurokinin γ. These sensory neuropeptides participate in the regulation of bone and cartilage homeostasis by exerting a trophic effect on cartilage and bone cells^38,39^. Muschter D et al.^39^ found that substance P can prevent age-related bone structural changes and has a synthetic metabolic effect on bone structure under pathological conditions. Family With Sequence Similarity 26 Member F (FAM26F, also known as CALHM6) is a recently discovered tetraspanin-like membrane glycoprotein that can be upregulated by stimuli like LPS, INF γ, and TNF α. CDC28 Protein Kinase Regulatory Subunit 2 (CKS2) exerts its function by binding to the catalytic subunit of cyclin-dependent kinase and can regulate cell proliferation as a target gene in the Wnt/β-catenin signaling pathway^40,41^. These study findings further validate the reliability of our analysis, however, further experimental validation is still required for the involvement of the 5 hub genes in proliferation and inflammation in osteoarthritis.

Furthermore, our analysis revealed that all five hub genes are positively correlated with Neutrophils, Activated CD4 T cell, Gamma delta T cell, and Regulatory T cell, while they are negatively correlated with CD56dim natural killer cell and Type 17 T helper cell. Recently, it has been established that bone and immune cells share the same progenitor cells and are influenced by the same cytokines^42^, indicating their functional interconnectedness. Immune cell regulation plays a crucial role in the onset and progression of OA^43^, including the regulation of dynamic balance and inflammation activation between bone cells and osteoclasts, such as macrophage polarization (M1/M2)^44^, Mast cells^45^, monocyte cells^46^, and the balance of Th1/Th2/Treg factors^47^. Previous studies have indicated that FAM26F can generate affinity interactions and potential synapses among various immune cells, including CD4^+^ T cells, CD8^+^ T cells, Natural killer cells, dendritic cells, and macrophages, potentially acting as a marker for IFN-γ driven immune responses^48^. CHI3L1 and MMP13 are pro-inflammatory biomarkers that indirectly control joint remodeling through protein hydrolysis of cell surface receptors or cytokine and receptor molecules^49,50^. The MMP13 precursor can be activated by neutrophil elastase^51^, while CHI3L1 is primarily derived from activated macrophages and chondrocytes, neutrophils, and some tissues and tumor cells, and can serve as a mitogen for fibroblasts^52,53^. The TAC1 gene encodes the neuropeptide substance P, which interacts with its coupled neurokinin receptors (NKRs) to elicit their activity, including NK1R, NK2R, and NK3R. NKRs are expressed on the surface of various cell types, including vascular and lymphatic endothelial cells, immune cells, fibroblasts, and neurons^54^. The study by Mou et al.^55^ suggests a significant association between CKS2-positive cancer-associated fibroblasts and poor prognosis, as well as low immune cell infiltration. Additionally, from an immune system perspective, assessing the degree of immune cell infiltration and identifying differences in the composition of immune cells associated with infiltration are crucial for elucidating the molecular pathogenesis of OA and developing new immunotherapeutic targets. In summary, the five hub genes may play a crucial role in OA by interlinking with PreHTC cells, other chondrocytes, and immune cells.

Additionally, molecular docking results revealed that CAY10603, Tenulin, T0901317, and Nonactin exhibited high binding activities to CHI3L1, indicating their potential as therapeutic drugs for OA. CAY10603, a specific inhibitor of histone deacetylase 6 (HDAC6), is a potential drug^56^. The expression level of HDAC6 was significantly decreased in OA samples, although the relationship between CAY10603 and OA has not been reported. However, a study by Yan J et al.^57^ found that inhibiting HDAC6 activity could block chondrocytes from releasing extracellular vesicles positive for LC3, effectively reversing pathological calcification and degradation of cartilage. Tenulin is an effective P-glycoprotein (P-gp) inhibitor^58^, and P-gp is associated with the development of methotrexate resistance in rheumatoid arthritis. T0901317 is a high-affinity liver X receptor (LXR) agonist that can enhance ABCA1 activity, thereby regulating the release of phospholipids in fibroblast-like synoviocytes from osteoarthritis joints^59^. Nonactin is a mitochondrial uncoupler that selectively induces apoptosis in tumor cells with β-chain protein mutations^60^. Furthermore, CHI3L1 can regulate cellular oxidative damage, apoptosis, and inflammatory activation through the activation of the Wnt/β-catenin signaling pathway^61,62^. In summary, CAY10603, Tenulin, T0901317, and Nonactin may have potential therapeutic effects for treating OA patients by targeting the CHI3L1. However, further experimentation is needed to confirm this.

Finally, this study conducted RT-qPCR experiments on clinical blood samples from OA patients and NC to investigate the expression levels of hub genes and their related TFs. The results revealed a significant increase in the mRNA expression levels of CHI3L1, CKS2, FAM26F, MMP13, TAC1, NFKB1, and RELA in the plasma of the OA group compared to the NC group, while the mRNA expression levels of CREB1, CREB3, and HDAC6 were significantly decreased. Transcription control analysis based on the NetworkAnalyst^33^ database suggested that CREB1 is a potential TF for CKS2 and TAC1, and NFKB1 and RELA are potential TFs for CHI3L1. Previous studies have indicated that CHI3L1 can enhance the activity of the NF-κB signaling pathway by binding to NFKB1^50^. Interestingly, the specific inhibitor CAY10603 of HDAC6 showed high binding activity with the molecular docking of CHI3L1^56^. Furthermore, another study demonstrated that the CREB site plays a key role in the SDF-1α mediated activation of TAC1^63^. These findings further support the potential of the five hub genes and their related TFs as diagnostic markers for OA.

Interestingly, our analysis revealed an unexpected finding: pre-hypertrophic chondrocytes (preHTC) from OA tissues showed a marked proliferative activity, contrasting with the senescence observed in healthy cartilage. This novel observation suggests a distinct role for preHTC in OA pathogenesis, potentially contributing to the disease’s progressive nature.

And compared with published studies on chondrocyte phenotype and genetic changes in OA development, our study screened five key hub genes (MMP13, FAM26F, CHI3L1, TAC1, CKS2) and related transcription factors by combining the latest bioinformatics data with human samples for validation and deeply explored their role in OA development, especially the new findings on prehypertrophic chondrocyte formation and chondrocyte proliferation, inflammation, and immune cell interactions. Although some of the results differ from previous studies, which may stem from differences in samples, methods, and data analysis, it is these differences that drive further exploration and understanding of OA pathogenesis.

There are also limitations to our study, that the plasma analysis of specific markers in OA patients is preliminary. To enhance the rigor and generalizability of our findings, we plan to expand our sample size in future studies to include patients from diverse geographic and ethnic backgrounds. Additionally, we aim to employ supplementary validation methods, such as Western blotting and ELISA, to corroborate the RT-qPCR results and ensure that the observed changes in gene expression accurately reflect the actual plasma concentrations of these markers. These additional experiments will provide more robust evidence to support our findings on OA biomarkers. In summary, the above-mentioned molecules may play a role in the interaction between PreHTC cells and other chondrocytes, as well as immune cells in OA. Further experimental validation is needed to understand the downstream pathways and metabolic changes of these molecules and their relationship with immune cells. However, our data analysis and RT-qPCR experiments show highly consistent results, confirming the reliability of our findings. Our results are expected to provide new insights into the immune regulation mechanisms of OA.

## Conclusion

The prehypertrophic chondrocytes (PreHTC) subpopulation plays a significant role in the development and progression of osteoarthritis (OA). Five hub genes may be involved in regulating cell proliferation and inflammation by interacting with PreHTC cells, other chondrocytes, and immune cells. Targeting these five hub genes could improve precision therapy and enhance early diagnosis and treatment of OA. Additionally, CAY10603, Tenulin, T0901317, and Nonactin have potential therapeutic effects for treating OA patients.

## Supplementary Information

Supplementary Information 1.

Supplementary Information 2.

Supplementary Information 3.

Supplementary Information 4.

## Data Availability

The dataset involved in the present study is available in the GSE152805, GSE19060, GSE82107, and GSE89408.
