# Supplementary material for: Identification of osteoarthritis-associated chondrocyte subpopulations and key gene-regulating drugs based on multi-omics analysis
Source: Sci Rep. 2025 Apr 11;15:12448. doi: 10.1038/s41598-025-90694-w (PMC11992032; doi:10.1038/s41598-025-90694-w)
Supplement: Supplementary file 1 — Supplementary Information 1. [file 41598_2025_90694_MOESM1_ESM.docx]

**Supplementary materials:**
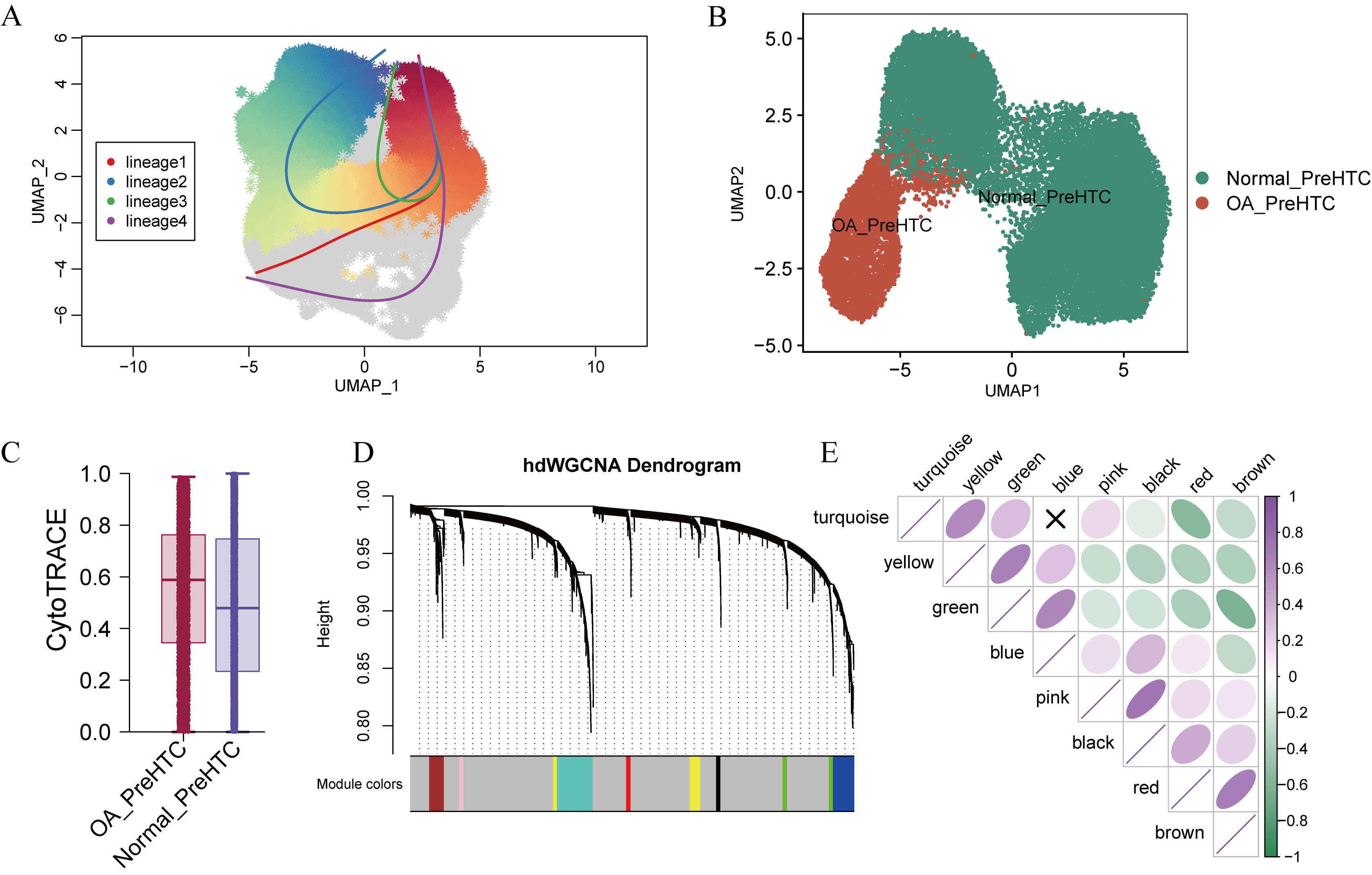


Supplementary figure 1. A. The pseudo-temporal differentiation trajectory of chondrocyte subpopulations; B. UMAP plot of PreHTC cell subpopulations; C. Differences in stem cell properties between OA_PreHTC and Normal_PreHTC cells; D. Dendrogram clustering analysis tree plot of hdWGCNA analysis; E. Heatmap of module correlations from hdWGCNA analysis.


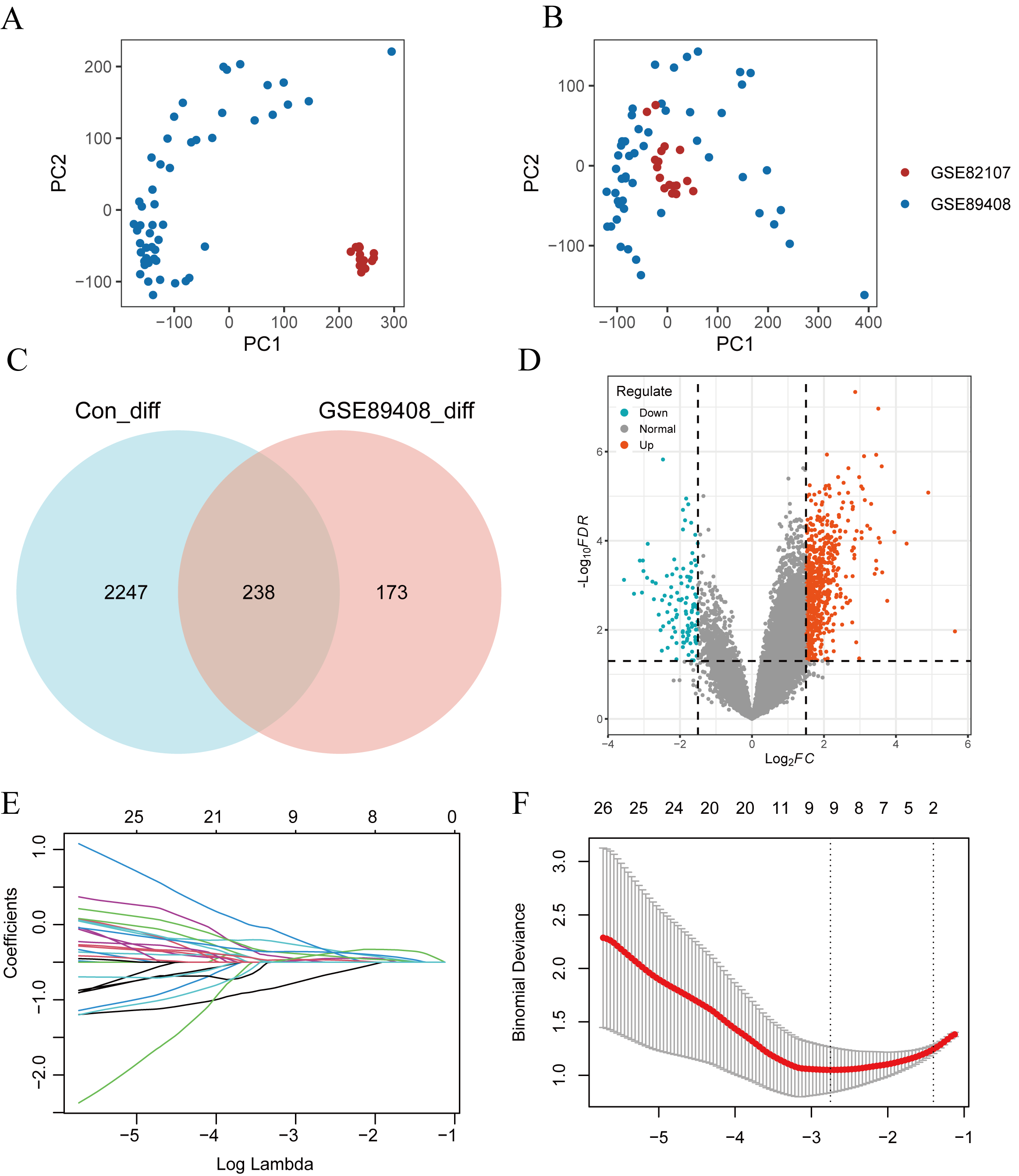


Supplementary figure 2. A. PCA plot before batch removal in the training set; B. PCA plot after batch removal in the training set; C. Venn diagram showing the intersection of differentially expressed genes (DEGs) between subgroups and GSE89408. This intersection resulted in 411 genes, which were further analyzed.; D. Volcano plot of all DEGs identified in GSE89408. Note that this plot includes all DEGs identified in the dataset, not just the 411 intersecting genes shown in C. The 411 genes were selected based on their statistical significance (adjusted p-value < 0.05) and fold change (|log2FC| > 1) in both the subgroup analysis and GSE89408 dataset；E. Lambda plot of logistic analysis used to select diagnostic marker genes from the 411 key differentially expressed genes; F. Selection of diagnostic marker genes from the 411 key differentially expressed genes using the Lasso algorithm.
