# Supplementary material for: Identification of osteoarthritis-associated chondrocyte subpopulations and key gene-regulating drugs based on multi-omics analysis
Source: Sci Rep. 2025 Apr 11;15:12448. doi: 10.1038/s41598-025-90694-w (PMC11992032; doi:10.1038/s41598-025-90694-w)
Supplement: Supplementary file 4 — Supplementary Information 4. [file 41598_2025_90694_MOESM4_ESM.docx]

### **AutoDockTools**

****URL：****[https://autodock.scripps.edu/](https://autodock.scripps.edu/)

### **PyMOL**

### ****URL：****[https://pymol.org/](https://pymol.org/)
